# JC virus in the pathogenesis of colorectal cancer, an etiological agent or another component in a multistep process?

**DOI:** 10.1186/1743-422X-7-42

**Published:** 2010-02-18

**Authors:** Tatiana R Coelho, Luis Almeida, Pedro A Lazo

**Affiliations:** 1 Instituto de Farmacologia e Terapêutica, Faculdade de Medicina, Universidade de Coimbra, Portugal; 2 Experimental Therapeutics and Translational Oncology Program, Instituto de Biología Molecular y Celular del Cáncer, Consejo Superior de Investigaciones Científicas (CSIC) - Universidad de Salamanca, Spain

## Abstract

JCV infection occurs early in childhood and last throughout life. JCV has been associated to colorectal cancer and might contribute to the cancer phenotype by several mechanisms. Among JCV proteins, particularly two of them, large T-antigen and agnoprotein, can interfere with cell cycle control and genomic instability mechanisms, but other viral proteins might also contribute to the process. Part of viral DNA sequences are detected in carcinoma lesions, but less frequently in adenomas, and not in the normal surrounding tissue, suggesting they are integrated in the host cell genome and these integrations have been selected; in addition viral integration can cause a gene, or chromosomal damage. The inflammatory infiltration caused by a local chronic viral infection in the intestine can contribute to the selection and expansion of a tumor prone cell in a cytokine rich microenvironment. JCV may not be the cause of colorectal cancer, but it can be a relevant risk factor and able to facilitate progression at one or several stages in tumor progression. JCV transient effects might lead to selective expansion of tumor cells. Since there is not a direct cause and effect relationship, JCV infection may be an alternative to low frequency cancer predisposition genes.

## 

Cancer is a multifactor disease that its progression is determined by several genetic alterations, which are most likely sequentially selected for their contribution to the tumor phenotype [1]. This phenotypic complexity makes difficult to determine the specific roles for biological agents that might be considered carcinogenic, and even more difficult to determine their causality, or implication at a particular stage in disease progression. Among the exogenous agents associated to cancer initiation or progression are chemicals, which are single molecules with a specific effect; and some infectious agents, including viruses and bacteria.

Infectious diseases are acquiring relevance as important pathogenic elements in human cancer, since almost one fifth of human cancers are associated with infectious agent, either bacteria or viruses, particularly in the gastrointestinal tract [2], but do not represent mainstream oncologic research. The development of several types of human cancers can be triggered by the exposure to different infectious agents, viruses or bacteria, such as Human Papilloma Virus (HPV) infection associated to cervical carcinoma [3], hepatitis B virus to liver carcinoma [4], Epstein-Barr virus to Burkitt lymphomas [5], HTVL-1 to ATL [6], *Helicobacter pylori *to gastric carcinoma [7] and more recently a human retrovirus, XMRV, has been associated to sporadic prostate cancer [8-10]. More recently, it has been reported the association between the development of lower gastrointestinal tract neoplasias and infectious agents [7], such as between colorectal cancer (CRC) and JCV infection. JCV is a virus very well adapted to humans, thus its widespread infection and adaptation to humans complicates the determination of its etiologic contribution to cancer development, and it has also been associated to some neurodegenerative diseases [11].

A chronic infection might be a non genetic alternative to cancer predisposing genes. The mechanistic role of infectious agents is even more complex, because they might contribute in several different ways to oncogenesis. Infections can also play a role at different stages in tumor progression, from initiators to promoters of the process, depending on the phenotypic aspect to which they can contribute and some might be transient or a consequence of the host response to chronic infection. Thus, causality of infectious agents in cancer is somewhat more difficult to demonstrate.

The viral contribution to oncogenesis has to be addressed by determining how the viral life cycle and the host response, including their alterations, can be integrated within the multistep process of tumor development. Viruses that are well adapted to humans can participate in oncogenesis process because they have not undergone a negative evolutionary pressure, since cancer develops at an age well above the median age of humans till the twentieth century, However, the benefits of interfering or preventing infectious agents implicated in cancer is very large as demonstrated in hepatic, gastric and cervical carcinomas [2].

Colorectal cancer (CRC) is the third most common tumor in women and the fourth in man; representing annually one million new cases worldwide, and more than 500.000 deaths are caused by this malignant disease [12]. Most colorectal cancers are sporadic in their origin and associated to different risk factors, such as a diet rich in fat and animal protein intake [13].

## JCV infection in human population

The extent of the exposure to JCV infection is indirectly known by seroprevalence rates detected in different populations throughout the world, ranging from 44 to 90% in the USA, UK, Germany, Brazil and Japan [7,14,15]; all indicating it is widespread and common. The infection may occur by fecal contamination and is usually persistent and sub clinical, but is reactivated under conditions of immunosuppression, such as in patients with AIDS, and JCV can also emerge from latency and become a lytic infection causing progressive multifocal leukoencephalopathy (PML) [16]. JCV infection occurs relatively early during childhood and seropositivity increases with age, and may be as high as 50% by the age of 10 [17]. Infection appears to occur through the gastrointestinal tract [18-20], but also it has been detected in the respiratory tract raising the possibility of an oral-fecal transmission [21,22]. The detection of antibodies from childhood to adults suggests that these antibodies are unlikely to be protective, however they are a clear indicator of exposure, probably multiple or sustained, to JCV. The persistence of seropositivity does not mean that individual patients have an active infection at the moment, or that harbor viral DNA or latent virus in some cells. The antibody mediated immune response is unlikely to be protective, and control of JCV infection is more probably performed by T-cell mediated responses, if successful [23]. It would be interesting to determine if tumor-infiltrating lymphocytes from colon carcinoma can recognize and react with JCV infected cells.

Alternatively, urinary secretion of JCV has been used as an alternative detection method. JCV was present in 24% of the samples out of 498 healthy individuals, and the proportion increased with age [24]. However, in immunocompromised patients there is a significant increase in JCV viruria, although it does not correspond with the level of immunosuppression, suggesting the presence of a subclinical infection that might require additional factors to be reactivated [25]. These data indicate that most people have been exposed for some time to this virus in order to generate an immune reaction. In cancer patients viruria was detected in up to 70 percent of the cases, but viral DNA could not be detected in carcinoma tissue; but this apparent discrepancy might have a methodological explanation, as primer selection may not be suitable in case of partial loss of viral DNA, or viruses might transiently contribute to a particular stage of tumor progression [14]; particularly since later several studies were able to detect viral DNA in carcinoma samples [15,26].

## The JC virus

The polyomaviruses family comprises SV40, JCV and BKV viruses, the primate SV40 virus is the reference member, while BKV and JCV are much poorly characterized, and mostly done with SV40 specific cross-reactive reagents [27]. Despite their isolation from humans, their role in human pathology is far from clear for both JCV and BKV. JCV is a nonenveloped virus with double-stranded DNA that forms minichromosomes with cellular histones. The JCV early region encodes two oncoproteins, large T antigen (T-Ag) and small t (t-Ag) antigen and the late region encodes three capsid proteins (VP1, VP2 and VP3) and a small regulatory protein (Agnoprotein). The viral DNA is packed as a non-enveloped virus in a virion composed of 72 pentamers of the major VP1 capsid protein, that also includes one unit of the minor VP2 and VP3 proteins [11]. These early viral proteins have a high transformation and oncogenic potential in experimental systems [28,29]. There are two possible outcomes to JCV infection: permissive cells, as oligodendrocytes, are able to support viral DNA replication resulting in a lytic infection; in nonpermissive cells, as those of the colorectal epithelium, resulting in silent or abortive infection, or probably in cell transformation and cancer [28]. Despite these evidences, the role of JCV in human malignancies, and of its oncoproteins in promoting transformation of cells in vitro and in vivo, is still far from clear. JCV does not infect experimental animals, and its roles have been implied by analogy to SV40 [30].

JCV is a human neurotropic polyomavirus, and neurological diseases, such as progressive multifocal leukoencephalopathy have been associated to JC virus [11,31]. Initial studies suggested that the transforming ability of JCV was limited to specific neural cell types, and that this property mapped to the noncoding regulatory sequence in the origin of DNA replication, but no neural tumor has been associated to JCV. However, JCV can also infect and transform different cell-types in culture and is highly oncogenic in several laboratory animal models [32]. Furthermore, there is evidence for the presence of the JCV genome in a broad range of human cell types and tissues. For example, the JCV genome has been detected in tonsillar stromal cells, B lymphoid cells, kidney epithelial cells, and upper and lower parts of the gastrointestinal tract, including the mucosa of the colon, which is considered as the natural human reservoir [18].

In addition, the JCV genome has variant forms which might behave differently from an oncogenic point of view. The Mad-1 strain, which lacks 98 nucleotides repeats in its transcriptional regulatory region, was the only one detected in colon carcinomas from California patients [33], but no functional study on the characteristics of this regulatory region has been performed. More recently in another study performed in Taiwan, most CRC cases appeared to have the JCV-CY genotype [15], which is also the dominant genotype in this population, although the Mad-1 strain was also detected [15]. Thus it is not clear if this strain association reflects the viral types circulating in different geographical areas, or if there are particular strains more strongly associated to carcinomas.

## JCV DNA in normal, benign and malignant colorectal lesions

JCV DNA sequences and proteins have been detected in a broad range of human tumors of glial and non-glial origin, including gliomas, ependymomas and medulloblastomas, as well as in several non-neural clinical specimens of upper and lower gastrointestinal tumors, such as colorectal cancer (CRC) [7], suggesting they can infect a wide range of cell types, but the role of JCV in human malignancies is still unclear. A very important issue is to determine whether the presence of JCV presents any difference between tumor of different grades and its normal surrounding mucosa. Very few studies have been performed in this context, but a picture of the situation is emerging from three independent studies in Japan [26], Taiwan [15] and South Korea [34]. Remnant JCV large- T DNA was detected in 28-80% of the cases, but protein was only detected in 16% of the carcinomas. Also the frequency is lower in benign adenomas, and negative in normal surrounding tissue [26]. DNA corresponding to agnoprotein and VP genes was not detected suggesting they were deleted [34]. More importantly in this study no viral DNA sequences were detected in the adjacent normal tissues [34]. The general findings are consistent among these three studies, and the variation in frequency is likely to be a consequence of the different primers used for viral DNA detection. However, a similar and earlier study in the USA was not consistent with this observation, but in that case there was no microdissection of the CRC biopsies and primers were different [14]. In none of these studies there is any indication about the JCV strain implicated.

The variability in JCV detection suggests that in an infected colon, in some cells there might be integration with partial loss of JCV DNA, which may have a pathogenic role in cancer development, probably permitting additional events that will lead to cancer progression by permitting selection of a cell subpopulation. When human CRC samples were grown as xenographs in nude mice that permit expansion of the cancer cell population, all of them resulted positive for JCV [18], suggesting that the cell subpopulation containing JCV might be selected for its growth and adaptation characteristics. In a way, this situation is reminiscent of what occurs in cervical carcinoma, in which most HPV infections and lesions regress, but some progress, and cervical carcinomas have remnant viral DNA coding for some viral proteins, E6 and E7, but the other HPV proteins are not expressed in these carcinomas [3]; these viral proteins participate in the oncogenic process, but clearly need additional, non virally related events [35,36].

## Multiple effects of JCV large T- antigen in host cells

JCV is able to translate its early proteins, namely LT-Ag, in order to trigger the progression of the cell cycle to the phase S in host-cell [29,37]. JCV LT-Ag has the ability to interact with p53; thus it is possible that in some cells this interference with p53 [38], which also interferes with viral replication [38], might allow the occurrence of additional genetic damage representing a step forward in colon carcinogenesis [39,40]. In addition, there are several additional mechanisms by which LT-Ag can also interfere with cellular functions. JCV LT-Ag can interact with proteins involved in cellular regulation such as IRS-1 (Insulin receptor substrate-1), a major protein of the insulin-like growth factor I receptor (IGF-IR) signaling pathway, which is activated and translocated to the nucleus in the presence of LT-Ag [41]. Activated IRS1, is an adaptor in the cell response to insulin, activating PI3K, implicated in cell survival [42], and proliferation signals [43]. Thus at the same time it will permit survival and expansion of a JCV containing subpopulation. Some polymorphisms of the IRS1 gene have been associated with an increased risk of colorectal cancer [44]. These signaling effects associated to LT-Ag can participate at any given stage of cancer progression, facilitating the expansion of a specific subpopulation, perhaps already pretumoral. LT-Ag can also inhibit homologous recombination directed DNA repair (HRR) causing DNA damage, mechanistically by its interaction with IRS1 [45], which also interacts with Rad51 at locations of damaged DNA [46,47], and thus may contribute to generate some genetic instability in cells containing JCV [48]. JCV also by a hit and run mechanism, that is a transient effect, is able to trigger genetic instability by interacting with p53 and β-catenin in colonic cells, which is detected only in the first seven days after infection [49]. LT-Ag also contributes to the stabilization of β-catenin by a novel mechanism mediated by the small GTPase Rac1 [50]. β-catenin is an integral component of the Wnt signaling pathway whose stabilization is associated with increased transcription of genes that regulate cellular proliferation, e.g., c-myc and cyclin D1, despite the fact that the functional consequence of this JCV interaction in cancer development remains to be elucidated. LT-Ag interacts with β-catenin [51], and β-catenin implication in colorectal cancer is well known [52,53]. This observation is supported by reports on the involvement of Wnt signaling pathway [54] and c-myc [55] in colorectal carcinogenesis. If the effect persists for some time in infected cells harboring JCV, they might contribute to expand a cell subpopulation that later might give rise to cancer.

## Effects of JCV agnoprotein in host cells

JCV expresses a small 71 aminoacids protein, known as agnoprotein, which is a regulatory protein that can repress the expression of p21WAF-1/Cip1, a regulator of the cell cycle that inhibits the activation of cyclin/CDK complexes and releases E2F transcription factor from phospho-Rb, thus agnoprotein removes an inhibitor of cell cycle progression. Consequently, p21WAF-1/Cip1 and E2F stimulate several proteins, the function of which is essential for cell cycle progression and rapid cell proliferation [56]. This role is in some aspects reminiscent of the action of E6 in cervical carcinogenesis, where the elimination of p53 also results in lack of induction of p21 [57,58], and permits cell cycle progression. Agnoprotein can also alter the expression of Ku70 and Ku80 [56], two proteins implicated in DNA repair [59,60], and thus indirectly contribute to a potential accumulation of genetic damage. Furthermore, agnoprotein can also interact with the YB-1 transcription factor [61], a factor that when is downregulated results in an induction of apoptosis [62] by regulating the mTOR/Akt pathway [63]. This YB-1 transcription factor can also modulate the response to the erbB2 receptor [64], and contribute prevention of premature senescence [65]. In addition YB-1 also controls some chemokine ligand [66] and metaloprotease gene expression, such as MMP13 [67], two types of proteins that can have an important role in carcinogenesis. YB-1 has been shown to have a predictive value in breast cancer patients identifying those with a poorer prognosis [68,69]. However, there is no systematic study of the presence and role of this viral protein, or of YB-1, at different stages of CRC progression. Agnoprotein has been shown to inhibit differentiation of oligiodendrocytes [70]; if a similar effect could be induced in colonic epithelium, it will be an additional contributing factor towards tumorigenesis. But a detailed study of agnoprotein presence in biopsies representing different stages of CCR has not yet been performed.

## Tumor immune microenvironment

Another important component of tumorigenesis is represented by the tumor microenvironment, where local cytokines that can play a stimulatory role. These cytokines may originate either in the tumor itself or in the local inflammatory infiltrate, and can activate JCV gene expression by a cis-acting transcription factor, Egr1; a factor which mostly activates the late promoter, and affects VP1 expression and viral replication [71]. The expression of this capsid protein can also trigger an inflammatory and immune reaction, with the corresponding local availability of several additional cytokines. This viral reactivation might also result in viral production, dissemination, and reinfection of neighboring cells. This mechanism could be important for maintenance or reactivation of a local subclinical infection. In this context it is important the recent observation that patients which have undergone liver transplantation have an increased risk of colorectal cancer due to reactivation of JCV, probably as a result of immunosuppressive treatment [72].

## Additional aspects in JCV associated oncogenesis

Another mechanism by which JCV can contribute to colorectal carcinogenesis might, in some aspects, be similar to the role of human papillomaviruses (HPV) in cervical carcinoma [35,36]. Viral DNA integration has only been partially addressed in cervical cancer associated to HPV [73], and the chromosomal location of viral integration sites coincides with those of fragile chromosome sites [74], and with translocation breakpoints already detected in other types of carcinomas [36,75], although the cellular genes affected are not known. If the JCV infection is persistent, then there is a good probability that JCV DNA might integrate in the host cell genome, and some of the viral DNA remains in a manner similar to what occurs with HPV in cervical carcinoma [74]. JCV DNA integration appears to be a common observation in CRC when this issue has been studied [26]; however its significance has not yet been properly addressed. This integration, if demonstrated, can explain why in some cases JCV is not detected. Integrated JCV DNA may be an important and irreversible component in the pathogenesis of CRC. In that way a cell population can harbor viral DNA from which some viral genes can be expressed with an altered regulation. The penetrance of the remaining viral gene and its level of expression might condition the risk of developing cancer. Also other viral genes might be lost, particularly those of the capsid, thus an immune reaction against them will not be effective.

## Roles that JCV can play in colorectal cancer

**Figure 1:**
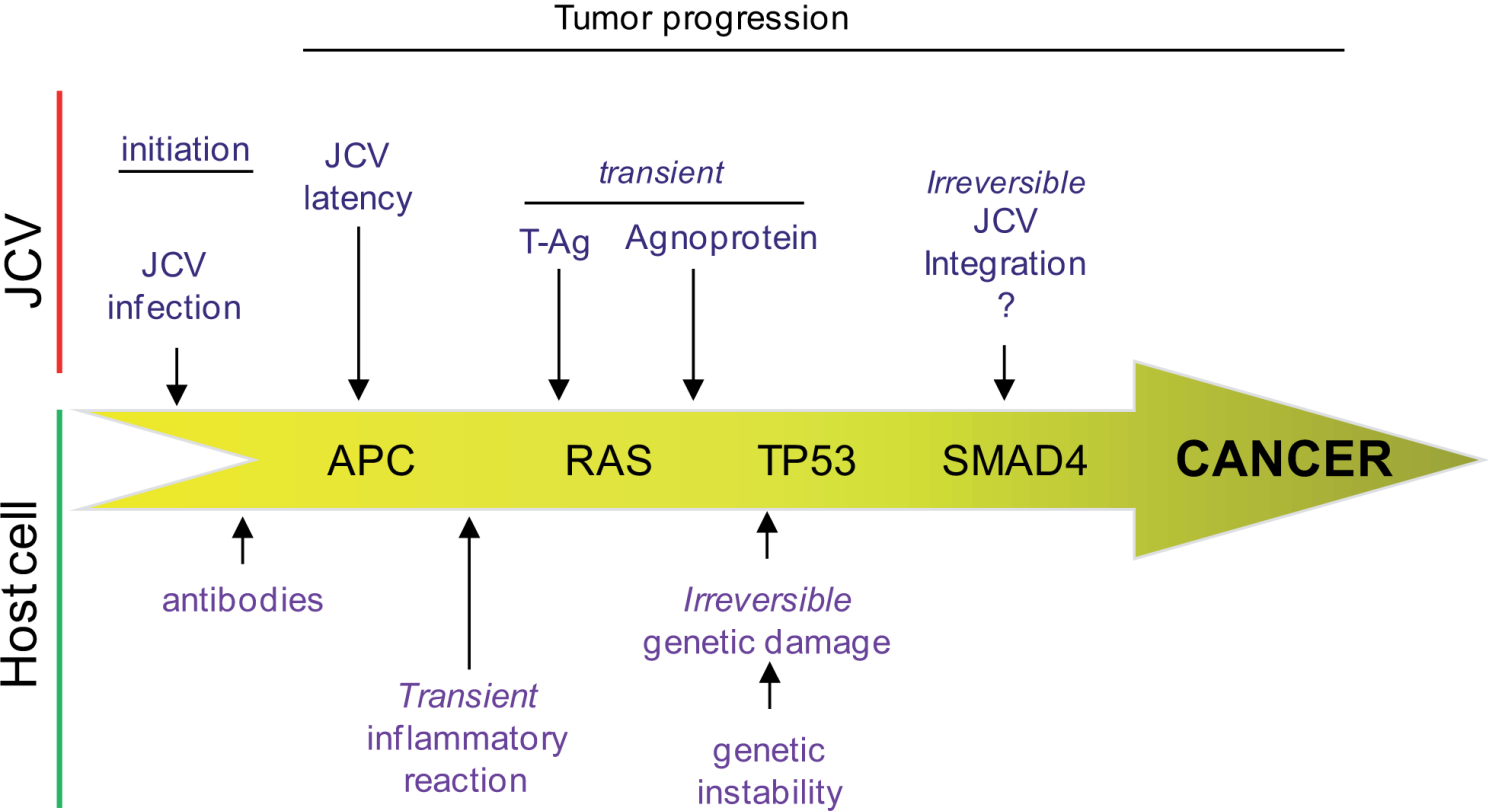
**Multistep colorectal carcinogenesis**. Diagram illustrating the different components by which JCV can participate in multistep colorectal oncogenesis, either in a transient or irreversible way.

The role of JCV in cancer does not fit within a classical concept of direct relation between cause and effect as it is for other infectious diseases and neither as a simple risk factor as applied to chemical or physical carcinogens. JCV might participate in different ways in the pathogenesis of colorectal cancer; both direct and indirect (Figure 1). This situation is a consequence of the complexity of the mechanisms contributing to cancer phenotype, which have many different phases, ranging from initiation, promotion, morphological progression with different biological characteristics, to tumor maintenance and dissemination. Thus, to pinpoint a unique mechanism of action for a virus represents a very simplistic approach to the problem. Some of the effects induced by JCV might be transitory and contribute to tumor progression at a particular stage of tumor progression. For example, a transitory genetic instability will permit generation of generic damage that might facilitate progression, but once it occurs, it is no longer needed. Other contributing factors are viral protein expression or viral DNA integration that might be important only at some steps in cancer progression. Also chronic infection is known to be a bad prognostic indicator due to the immune cell infiltration and generation of a microenvironment very rich in cytokines, which can promote expansion of premalignant or malignant cells, not necessarily with JCV, that will facilitate cancer cell growth and dissemination. Some of the effects might be transient and this will further complicate establishing a direct relation between JCV and CRC. In any case there is no evidence, epidemiological or physiopathological, to rule out a role for JCV in colorectal carcinogenesis, even if its role is still undefined; but on the contrary there is enough information pointing to roles that remain to be conclusively established.

## Outstanding issues and future prospects

The role of any virus as a causal agent in cancer should not be considered simply as a direct cause-effect. The virus should be considered as a complex agent with potentially multiple and varying effects. In order to properly establish if JCV is indeed an important risk factor for colorectal cancer there are several issues that need to be properly addressed.

1. Establish conclusively that CRC patients, in a significant number of cases, have been exposed to this virus, and of which an indirect marker will be the presence of specific antibodies.

2. Improve detection of viral DNA by using standardized sets of primers for each of the relevant viral genes, as well as strain identification. This should also contribute to establish the potential and irreversible integration of remnant JCV DNA in cancer cells, and permit identification of significant differences among malignant, benign or normal surrounding tissue.

3. Correlate JCV DNA presence with other mutations known to sequentially occur in CRC, and its association with other known risk factors.

4. Identification of viral protein expression at different stages of colorectal cancer progression. For this aim development of better specific antibodies for JCV proteins are necessary.

5. Characterize the host immune response to JCV in order to manipulate it and develop strategies to eradicate the virus from the human population. In particular determine the role T-cell responses, since the natural antibody response does not seem to be protective.

## Competing interests

The authors declare that they have no competing interests.

## Authors' contributions

TRC, LA and PAL conceived the study, and participated in its design and coordination. All authors read and approved the final manuscript.
